# Supplementary material for: Phospholipid Scramblase 4 (PLSCR4) Regulates Adipocyte Differentiation via PIP3-Mediated AKT Activation
Source: Int J Mol Sci. 2022 Aug 29;23(17):9787. doi: 10.3390/ijms23179787 (PMC9456373; doi:10.3390/ijms23179787)
Supplement: Supplementary file 1 [file ijms-23-09787-s001.zip › ijms-1830638-supplementary.pdf]

Supplementary Figures

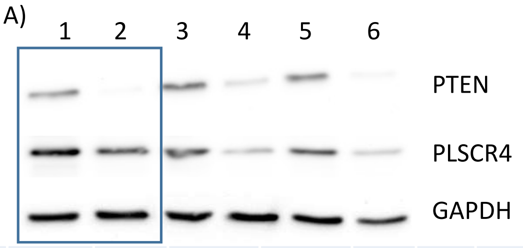

Lane	cell strain	passage	generation	siRNA	GAPDH	PLSCR4	PLSCR4/GAPDH
1	SVF#3	16	24.5	control	16706.36	7745.81	0.46
2	SVF#3	16	24.5	PTEN	18823.89	5175.98	0.27
3	SVF#6	11	18	control	16115.07	4561.98	0.28
4	SVF#6	11	18	PTEN	19668.43	1611.84	0.08
5	SVF#9	13	18	control	16679.77	4078.91	0.24
6	SVF#9	13	18	PTEN	10680.05	1212.31	0.11

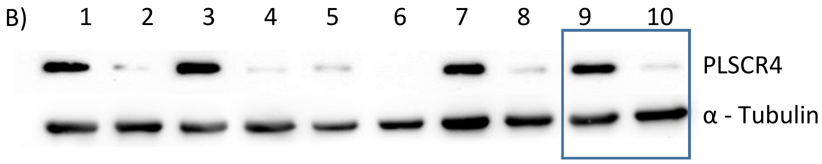

Lane	cell strain	passage	generation	siRNA	α-Tubulin	PLSCR4	PLSCR4/alpha Tubulin
1	SVF#9	14	19	control	7759.175	16144.803	2.081
2	SVF#9	14	19	PLSCR4	7404.296	663.698	0.090
3	SVF#6	16	25.5	control	5638.983	17495.167	3.103
4	SVF#6	16	25.5	PLSCR4	6615.811	413.092	0.062
7	SVF#6	14	20.5	control	9810.175	15295.045	1.559
8	SVF#6	14	20.5	PLSCR4	8292.711	1118.062	0.135
9	SVF#13	15	19.5	control	6898.811	15133.045	2.194
10	SVF#13	15	19.5	PLSCR4	9427.004	802.991	0.085

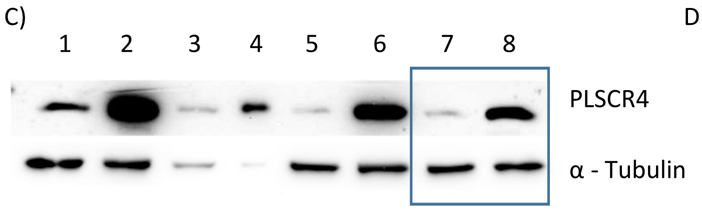

Lane	cell strain	passage	generation	plasmid	α-Tubulin	PLSCR4	PLSCR4/α-Tubulin
1	SVF#14	8	9.5	control	9354.761	10922.510	1.168
2	SVF#14	8	9.5	PLSCR4	7154.054	52679.082	7.364
5	SVF#15	5	6	control	6454.104	1175.477	0.182
6	SVF#15	5	6	PLSCR4	6818.640	32078.927	4.705
7	SVF#16	5	6	control	6306.276	1836.033	0.291
8	SVF#16	5	6	PLSCR4	6352.861	19566.823	3.080

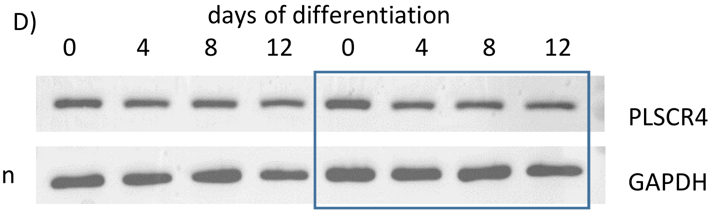

days of differentiation	cell strain	passage	generation	GAPDH	PLSCR4	PLSCR4/GAPDH
0	LipPD1	22	30.5	12191.368	17447.966	1.431
4	LipPD1	22	30.5	10638.539	12533.773	1.178
8	LipPD1	22	30.5	12003.610	13491.187	1.124
12	LipPD1	22	30.5	9050.468	10903.823	1.205
0	LipPD1	24	33.5	13009.196	19438.087	1.494
4	LipPD1	24	33.5	11892.660	11519.359	0.969
8	LipPD1	24	33.5	13385.610	12604.187	0.942
12	LipPD1	24	33.5	11715.953	11478.945	0.980

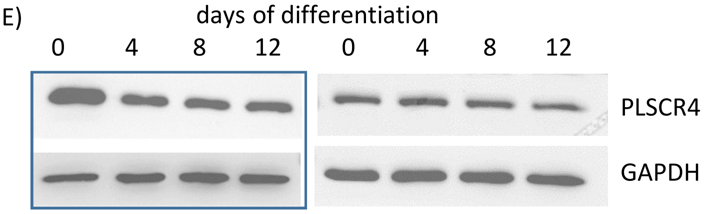

days of differentiation	cell strain	passage	generation	GAPDH	PLSCR4	PLSCR4/GAPDH
0	SVF#1	10	14	12735.6	13219.6	1.038
4	SVF#1	10	14	17489.8	4388.1	0.251
8	SVF#1	10	14	19831.6	4141.2	0.209
12	SVF#1	10	14	18081.9	4010.4	0.222
0	SVF#1	12	17	31265.5	17218.9	0.551
4	SVF#1	12	17	36189.9	18895.2	0.522
8	SVF#1	12	17	35031.3	16337.4	0.466
12	SVF#1	12	17	33814.4	13687.5	0.405

Figure S1: Western blots from PTEN and PLSCR4 knockdown/overexpression adipocyte progenitor cells (APCs). A) Western blots and densitometric analysis of control and PTEN siRNA transfected APCs. PTEN and PLSCR4 protein were normalized to GAPDH. B) Western blots and densitometric analysis of control and PLSCR4 siRNA transfected APCs. PLSCR4 protein was normalized to α-Tubulin. Due to low basal PLSCR4 expresssion lanes 5 and 6 were excluded from analysis. C) Western blots and densitometric analysis of control and PLSCR4 plasmid (PLSCR4 overexpression) –transfected APCs. PLSCR4 protein was normalized to α-Tubulin. Due to unequal expression of housekeeping gene α – Tubulin lanes 3 and 4 were excluded from analysis. D) Western blots and densitometric analysis of PLSCR4 during adipogenesis in LipPD1 cells. PLSCR4 protein was normalized to GAPDH. E) Western blots and densitometric analysis of PLSCR4 during adipogenesis in APCs. PLSCR4 protein was normalized to GAPDH. Western blot images presented in Figures 2, 3 and 4 were reused (indicated with blue boxes).

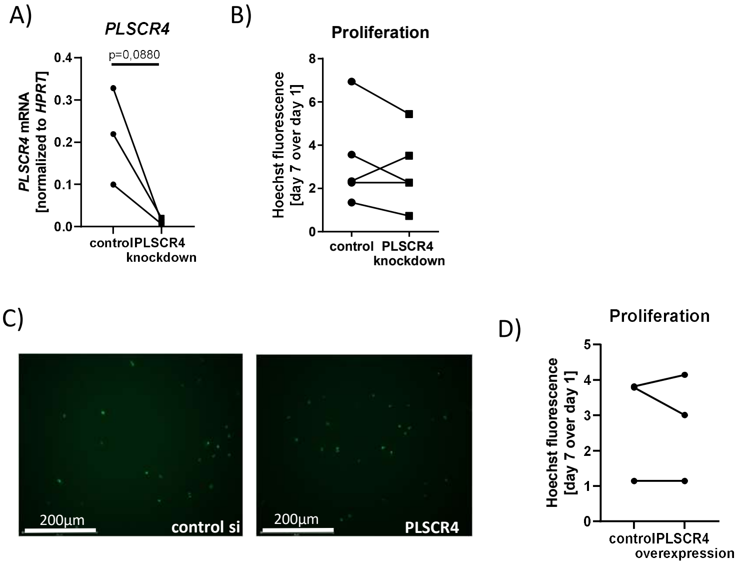

Figure S2. Knockdown/overexpression efficiencies in adipocyte progenitor cells (APCs). and effects on proliferation. A) qPCR of control and PLSCR4 siRNA transfected APCs: *PLSCR4* mRNA expression was downregulated 94.0% ± 29.9% (normalized to *HPRT*, n = 3, *p* = 0.0880). B) Hoechst nuclei staining of control and PLSCR4 siRNA: transfected APCs 7 days after transfection no significant difference in proliferation of APCs was observed (13.7% ± 14.8%, n = 5, *p* = 0.4044). C) Proliferation marker Ki-67 (green) immunofluoresence staining in control and PLSCR4 KD SVF cells showed no difference. D) Hoechst nuclei staining of control and PLSCR4 plasmid transfected APCs 7 days after transfection: no significant difference in proliferation of APCs was observed (5.2% ± 11.2%, n = 3, *p* = 0.6910). Matched results were visualized via lines between data points (control versus knockdown/overexpression). p-values were determined via paired t test (∗∗∗p < 0.001).

A)

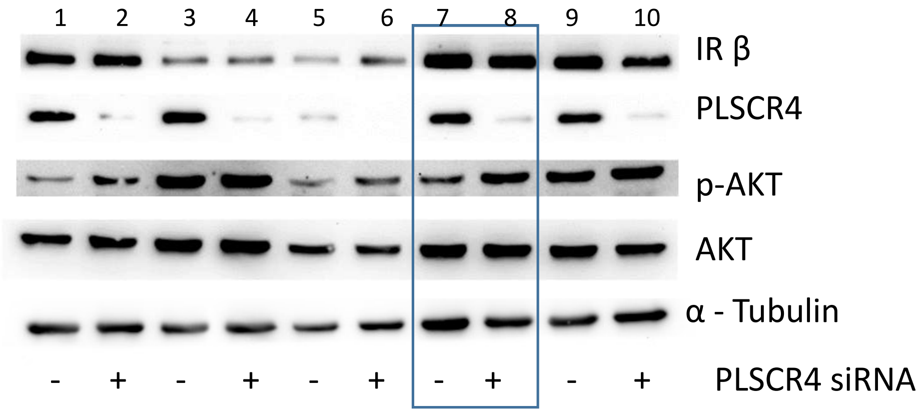

Lane	cell strain	passage	generation	siRNA	α-Tubulin	IR β	Akt	P-AKT	PLSCR4	IR β/α-Tubulin	PLSCR4/α-Tubulin	p-Akt/ Akt
1	SVF#9	14	19	control	7759.18	3565.67	15998.24	3186.91	16144.8	0.460	2.081	0.199
2	SVF#9	14	19	PLSCR4	7404.3	3408.67	15766.36	7419.54	663.7	0.460	0.090	0.471
3	SVF#6	16	25.5	control	5638.98	941.82	20374.14	14694.51	17495.17	0.167	3.103	0.721
4	SVF#6	16	25.5	PLSCR4	6615.81	892.28	21104.09	15507.85	413.09	0.135	0.062	0.735
5	SVF#10	16	20.5	control	4429.79	338.21	11146.49	2683.81	1064.6	0.076	0.240	0.241
6	SVF#10	16	20.5	PLSCR4	5906.15	1033.23	10280.61	6045.71	0	0.175	0.000	0.588
7	SVF#6	14	20.5	control	9810.18	6833.62	17256.51	6604.66	15295.05	0.697	1.559	0.383
8	SVF#6	14	20.5	PLSCR4	8292.71	4595.96	18066.29	13509.2	1118.06	0.554	0.135	0.748
9	SVF#13	15	19.5	control	6898.81	5076.21	16970.17	13363.95	15133.05	0.736	2.194	0.787
10	SVF#13	15	19.5	PLSCR4	9427	2807.55	15004.51	15031.08	802.99	0.298	0.085	1.002

B)

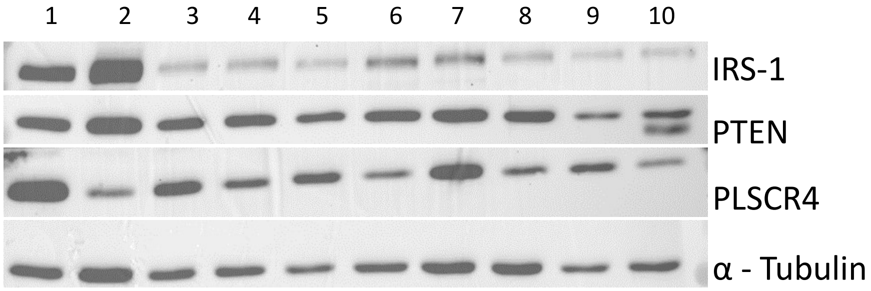

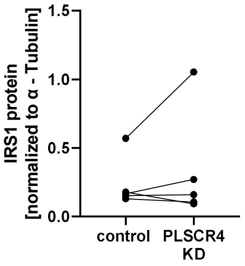

Lane	cell strain	passage	generation	siRNA	α-Tubulin	IRS1	PTEN	IRS1/α-Tubulin	PTEN/α-Tubulin	PLSCR4/α-Tubulin
1	SVF#10	p19	g23	control	17007.48	9695.823	10934.702	1.13	0.64	1.18
2	SVF#10	p19	g23	PLSCR4	24001.966	25294.543	20486.723	0.81	0.85	0.10
3	SVF#6	p12	g15,5	Control	11364.045	1737.82	8509.388	4.90	0.75	0.63
4	SVF#6	p12	g15,5	PLSCR4	11775.217	1876.062	9726.803	5.18	0.83	0.25
5	SVF#11V	p11	g14,5	Control	7151.61	1180.749	5282.539	4.47	0.74	0.61
6	SVF#11V	p11	g14,5	PLSCR4	11135.095	3013.69	9937.095	3.30	0.89	0.19
7	SVF#8	p16	g29,5	control	17052.945	3039.69	13635.995	4.49	0.80	0.69
8	SVF#8	p16	g29,5	PLSCR4	16225.53	1487.991	9278.803	6.24	0.57	0.14
9	SVF#6	p17	g22,5	Control	6825.782	883.092	2461.861	2.79	0.36	0.44
10	SVF#6	p17	g22,5	PLSCR4	10277.217	1085.335	4046.539	3.73	0.39	0.14

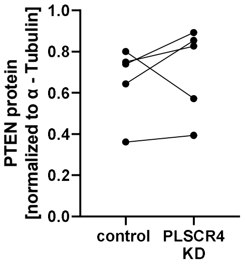

Figure S3. Western blots of PLSCR4 knockdown (KD) adipocyte progenitor cells (APCs). A) Insulin receptor β subunit (IR β),) and PLSCR4 were normalized to α-Tubulin. Phosphorylated AKT (pAKT T308) was normalized to total AKT protein. AKT phosphorylation in PLSCR4 KD cells was increased by 52.0% ± 13.6% compared to control cells (n = 5, p = 0.0186). *IR* β subunit expression was variable between control cells and *PLSCR4* KD cells and showed no regulation (n = 5, p = 0.3283). B) Insulin receptor substrate 1 (IRS-1), PTEN and PLSCR4 were normalized to α-Tubulin. IRS-1 (n = 5, p = 0.3931) and PTEN (n = 5, p = 0.5500) expression was not significantly different between control and PLSCR4 KD cells. Western blot images presented in Figure 5 were reused (indicated with blue boxes).

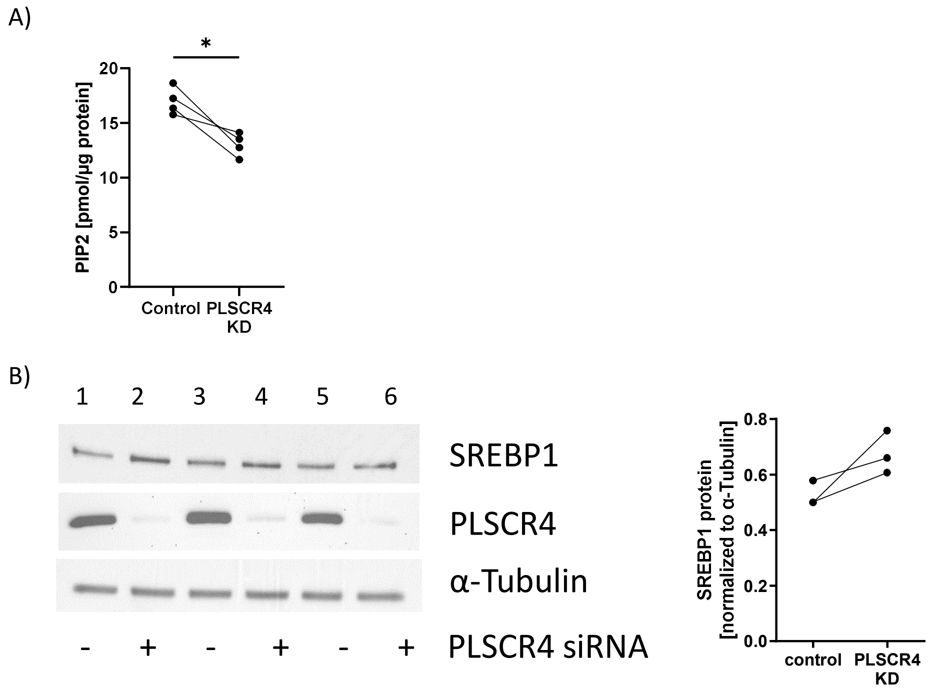

Lane	cell strain	passage	generation	siRNA	α-Tubulin	PLSCR4	SREBP1	PLSCR4/α-Tubulin	SREBP1/α-Tubulin
1	SVF#1	22	29	control	17381.1	28227.7	8693	1.62	0.5
2	SVF#1	22	29	PTEN	18438	1817.7	13988.7	0.1	0.76
3	SVF#9	11	36.5	control	17579.8	25842.3	10168.6	1.47	0.58
4	SVF#9	11	36.5	PTEN	18445.2	2092.1	12164.9	0.11	0.66
5	SVF#10	31	36	control	17358.3	13942.5	8705.4	0.8	0.5
6	SVF#10	31	36	PTEN	18349.9	346.2	11144	0.02	0.61

Figure S4. A) Amount of PIP2 in control and PLSCR4 KD APCs as determined via high pressure liquid chromatography-mass spectrometry (HPLC-MS) . PIP2 levels decreased by 23.1 ± 4.7% in PLSCR4 KD cells ($n = 4$, $p = 0.0215$). B) Western blots of PLSCR4 knockdown (KD) adipocyte progenitor cells (APCs). Sterol regulatory element-binding protein 1 (SREBP1) and PLSCR4 were normalized to α-Tubulin. SREBP1 expression was elevated in PLSCR4 KD cells by 28.9% ± 11.6% compared to control cells ($n = 3$, $p = 0.116$). p-values for the experiments were determined via paired t-test ($*p < 0.05$). PLSCR4, phospholipid scramblase 4.

Supplementary Tables

Table S1: Distance matrix of the PLSCR genes from Human, Chimpanzee, and Mouse species.

**PLSCR1**			**PLSCR2**			**PLSCR3**			**PLSCR4**			**PLSCR5**			
**Human**	**Mouse**	**Chimpanzee**	**Human**	**Mouse**	**Chimpanzee**	**Human**	**Mouse**	**Chimpanzee**	**Human**	**Mouse**	**Chimpanzee**	**Human**	**Mouse**	**Chimpanzee**	
0	0	0	16	24.68	24.57	46.4	45.8	49.49	51.63	50.5	48.99	40.38	42.96	45.19	**PLSCR1**
			0	0	0	46.67	49.64	48.71	50	52.88	51.97	38.67	45.98	45.34	**PLSCR2**
						0	0	0	59.71	61.19	59.15	52.85	53.28	52.94	**PLSCR3**
									0	0	0	56.93	57.03	60.08	**PLSCR4**
												0	0	0	**PLSCR5**

The distance is calculated based on the formula, Distance Score = m/(npos + gaps*gap_penalty), where m = score of matches (1 for an exact match, 0 for no match), npos = the number of positions included in m, gaps = number of gaps in the sequences, and gap_penalty = the score given to a gapped position (value set as Zero). The distances are expressed in terms of the number of substitutions per 100 amino acids and a score of Zero indicates 100 % similarity.

Table S2: Primers used for RT-qPCR

Gene	Forward	Reverse	Probe
hTBP	TTG TAA ACT TGA CCT AAA GAC CAT TGC	TTC GTG GCT CTC TTA TCC TCA TG	AAC GCC GAA TAT AAT CCC AAG CGG TTT G
hHPRT	GGC AGT ATA ATC CAA AGA TGG TCA A	GTC TGG CTT ATA TCC AAC ACT TCG T	CAA GCT TGC TGG TGA AAA GGA CCC C
hPTEN	TGTAAAGCTGGAAAGGGACGA	GGAATAGTTACTCCCTTTTTGTCTC	
hPLSCR4	CAGTACAACTAGACCCGGCG	CCAGGGGGTCCTGGTAAAAA	
mHprt	TCCTCCTCAGACCGCTTTT	CATAACCTGGTTCATCATCGC	
mTbp	GGGTATCTGCTGGCGGTTT	TGAAATAGTGATGCTGGGCACT	
mPTEN	TCCCAGACATGACAGCCATC	TGCTTTGAATCCAAAAACCTTACT	
mPlscr4	AAATGTCAGGTCTGGTCCCCA	AGAAGGAGAGGCAACTGGTC	

Table S3: Western blot (Wb) antibodies

Primary antibody	Dilution	Distributor	Cat.No.
AKT antibody Rabbit polyclonal Ab	1:1000 TBS-T 5%BSA (Wb)	CST	#9272
Phospho-AKT (Thr308) (224F9) Rabbit mAb	1:1000 TBS-T 5%BSA (Wb)	CST	#4056
alpha Tublin (11H10) Rabbit mAb	1:2000 TBS-T 5%BSA (Wb)	CST	#2125
GAPDH (6C5) Mouse mAb	1:50,000 TBS-T 5% milk (Wb)	Merck	MAB374
PTEN (138G6) Rabbit map	1:1000 TBS-T 5%BSA (Wb)	CST	#9559
Insulin Receptor beta (4B8) Rabbit mAb	1:500 TBS-T 5%BSA (Wb)	Cell Signaling	3025S
IRS-1 antibody Rabbit mAb	1:1000 TBS-T 5%BSA (Wb)	Cell Signaling	2382S
Anti PLSCR4 antibody Rabbit polyclonal Ab	1:1000 TBS-T 5%BSA (Wb)	Abcam	ab 233005

Secondary antibody	Dilution	Distributor	Cat.No.
Polyclonal goat anti-rabbit immunoglobulin/HRP	1:2000 TBS-T 5% milk (Wb)	Dako	P0447
Polyclonal goat anti-mouse immunoglobulin/HRP	1:2000 TBS-T 5% milk (Wb)	Dako	P0448
